# Immunogenicity and safety of HBAI20 Hepatitis B vaccine in non‐responders: Double‐blinded, randomised, controlled phase 2 trial

**DOI:** 10.1111/liv.14939

**Published:** 2021-05-24

**Authors:** Özgür M. Koc, Philippe de Smedt, Cécile Kremer, Geert Robaeys, Pierre van Damme, Niel Hens, Jorge Almeida, Frank Falkenberg, Paul Savelkoul, Astrid Oude Lashof, the BE RESPONDER Study Group

**Affiliations:** ^1^ Department of Medical Microbiology School of NUTRIM Maastricht UMC+ Maastricht The Netherlands; ^2^ Department of Gastroenterology and Hepatology Ziekenhuis Oost‐Limburg Genk Belgium; ^3^ Faculty of Medicine and Life Sciences Hasselt University Hasselt Belgium; ^4^ Centre for the Evaluation of Vaccination Vaccine & Infectious Disease Institute Antwerp University Antwerp Belgium; ^5^ Data Science Institute I‐BioStat Hasselt University Hasselt Belgium; ^6^ Department of Gastroenterology and Hepatology KU Leuven Leuven Belgium; ^7^ Centre for Health Economic Research and Modelling Infectious Diseases Vaccine & Infectious Disease Institute Antwerp University Antwerp Belgium; ^8^ CyTuVax B.V Maastricht The Netherlands; ^9^ CIRES GmbH Dortmund Germany; ^10^ Department of Medical Microbiology & Infection Control Amsterdam University Medical Centers VUMC Amsterdam The Netherlands

**Keywords:** adjuvant, HBAI20, hepatitis B vaccine, immunogenicity, non‐responder

## Abstract

**Background & Aims:**

Approximately 5%–10% of the general population respond inadequately to licensed recombinant hepatitis B vaccines. We assessed the immunogenicity and safety of a new HBAI20 vaccine, consisting of a new AI20 adjuvant (20‐µg recombinant human IL‐2 attached to 20‐µg aluminium hydroxide) in combination with HBVaxPro®‐10 µg.

**Methods:**

In a double‐blinded, randomised, controlled phase 2 trial, 18‐ to 59‐year‐old healthy non‐responders (titre <10 mIU/ml after three or more doses of hepatitis B vaccine) were assigned (3:1 ratio) to receive either HBAI20 vaccine or HBVaxPro®‐10 µg in a 0, 1 and 2‐month schedule. The primary outcome was seroprotection (titre ≥ 10 mIU/ml) measured 1‐3 months following the third vaccination.

**Results:**

A total of 133 participants were randomised to receive either HBAI20 vaccine (n = 101) or HBVaxPro®‐10 µg (n = 32). In the modified intention‐to‐treat analysis, the seroprotection rate after the third vaccination was 92.0% (80/87) in the HBAI20 group and 79.3% (23/29) in the HBVaxPro®‐10‐µg group, *P* = .068. Using a generalised linear mixed model to adjust for stratification factors, a higher odds of seroprotection with HBAI20 vaccine was shown (adjusted odds ratio = 3.48, *P* = .028). Frequency of mild and moderate local adverse events was greater in the HBAI20 group than in the HBVaxPro®‐10 µg. Rates of severe local adverse events and systemic adverse events were low and similar in both groups.

**Conclusions:**

In this group of hepatitis B vaccine non‐responders, the HBAI20 vaccine demonstrated a higher seroprotection rate when adjusting for stratification factors and a similar safety profile compared to the licensed recombinant HBVaxPro®‐10 µg.

**Abbreviations**
anti‐HBc: hepatitis B core antibodies
anti‐HBs: antibodies against HBsAg
CI: confidence interval
FCS: fully conditional specification
GMCs: geometric mean concentrations
HBAI20: consisting of a new AI20 adjuvant (20‐µg recombinant human IL‐2 attached to 20‐µg aluminium hydroxide) in combination with HBVaxPro®‐10 µg
HBsAg: hepatitis B surface antigen
HBV: hepatitis B virus
HCV: hepatitis C virus
mITT: modified intention‐to‐treat analysis
MPL: 3‐O‐desacyl‐4′‐monophosphoryl lipid A
OR: odds ratio
SD: standard deviation
ULN: upper limit of normal

## 1 INTRODUCTION

Hepatitis B virus (HBV) infection is one of the most common and serious infectious diseases globally. It is estimated that one third of the world's population has been exposed to HBV, which has resulted in 257 million chronic HBV carriers worldwide.^1^ The economic burden of HBV infection is substantial because of the high morbidity and mortality associated with cirrhosis, hepatocellular carcinoma and liver failure.^2^

HBV is up to 100 times more infectious than HIV.^1^ As such, the development of a hepatitis B vaccine has been a major breakthrough in the global effort to eradicate HBV infection.^3^ Current licensed HBV vaccines are produced in *Saccharomyces cerevisiae* and are composed of hepatitis B surface antigen (HBsAg) which is adsorbed to aluminium hydroxide or aluminium phosphate.^4^ Despite their proven immunogenicity and safety, currently available recombinant hepatitis B vaccines are unable to induce an adequate immune response in 5%–10% of the general adult population.^5^ Persons lacking this antibody immune response (anti‐HBs level < 10 mIU/ml measured at 1‐3 months after completion of the hepatitis B vaccination schedule) are referred to as non‐responders.^5^ The standard of care for non‐responders consists of revaccination with currently licensed hepatitis B vaccines and is associated with a seroprotection rate of 50%–69% after three doses.^6^, ^7^, ^8^, ^9^, ^10^ Thus, there is a need for a more immunogenic vaccine in defined groups such as healthy non‐responders to recombinant hepatitis B vaccines.

Recently, various strategies have been developed to elicit protective anti‐HBs levels in hepatitis B vaccine non‐responders: intradermal vaccination, increased antigen dose, adjuvanted vaccines and others (eg, Twinrix®, Sci‐B‐Vac®, DNA vaccines, polypeptide micelle vaccines).^11^, ^12^ Among healthy adult non‐responders to primary hepatitis B vaccination, a previous double‐blinded, randomised, controlled trial in China demonstrated seroprotection in 92.1% (394/428), 87.1% (371/426) and 83.0% (180/217) with hepatitis B vaccines, respectively, containing 60‐, 30‐ and 10‐µg HBsAg.^12^ Moreover, two single‐blinded, randomised, controlled trials in healthy non‐responders showed a higher immunogenic response with adjuvanted vaccines, one with Fendrix® and one with Heplisav‐B®.^8^, ^10^ Among a total of 82 participants, the seroprotection rate was 97.5% in the group that received three doses of Fendrix® compared to 68.0% with three doses of Engerix‐B®.^8^ The observed higher seroprotection rate with Fendrix® was not only linked to the adjuvant 3‐O‐desacyl‐4′‐monophosphoryl lipid A (MPL) but also to the higher antigen content, ie, 40‐µg HBsAg in Fendrix® vs. 20 µg in Engerix‐B®.^8^ The study of Halperin et al^10^ was conducted among 35 non‐responders, and seroprotection was found in 88.9% and 66.7% after two doses with Heplisav‐B® and Engerix‐B®, respectively. At present, Fendrix® is only licensed for individuals with renal alteration, while Heplisav‐B® was never licensed in Europe. Most pivotal studies for the license application of Fendrix® and Heplisav‐B® have been performed on healthy vaccine naive adults and in the case of Fendrix® also on (pre‐) dialysis patients.^13^, ^14^, ^15^, ^16^, ^17^, ^18^, ^19^, ^20^, ^21^, ^22^, ^23^, ^24^, ^25^ More research on hepatitis B vaccines among the group of healthy non‐responders is therefore warranted.

Since the available recombinant hepatitis B vaccines all have an aluminium‐based adjuvant, we report for the first time the immunogenic properties of a cytokine‐based adjuvant in a well‐designed double‐blinded, randomised, controlled trial in healthy adult non‐responders. In a phase 1 trial, the new AI20 adjuvanted (HBAI20) vaccine was shown to be safe, well‐tolerated and immunogenic in healthy naive and non‐responding adults.^26^ The current study describes a phase 2 trial to evaluate in healthy non‐responders the immunogenicity and safety of the new HBAI20 hepatitis B vaccine compared to the licensed HBVaxPro®‐10‐µg vaccine.

## 2 MATERIALS AND METHODS

### 2.1 Study design

The study was a phase 2, double‐blinded (participant and investigator), randomised, controlled, multicentre trial. The protocol (in accordance with the CONSORT statement) was approved by the local ethics committee and was conducted in accordance with the guidelines of the Declaration of Helsinki and its amendments and in accordance with good clinical practice and local laws.

### 2.2 Participants

The participants were hepatitis B vaccine non‐responders, ranging in age from 18 to 59 years. Non‐responders were defined as subjects with documented three or more hepatitis B vaccinations and documentation of hepatitis B surface antibody (anti‐HBs) level < 10 mIU/ml measured within 1‐3 months after the last vaccination.^5^

Non‐responders were identified from Occupational Health Services located in the Netherlands and Belgium and subsequently invited for enrolment in one of the trial sites: Maastricht UMC+ (the Netherlands), Antwerp University (Belgium) or Hospital East‐Limburg (Belgium). The following exclusion criteria were used in the study: anti‐HBs level ≥ 10 mIU/ml, HBsAg positivity, positive for hepatitis B core antibodies (anti‐HBc), positive for HCV antibodies, positive for HIV antibodies, any infectious disease at the time of screening or enrolment, known or suspected immune deficiency, known or suspected disease that influences the immune system (eg, cancer and transplantation recipients), known or suspected allergy to any of the vaccine components, dialysis patients, history of unusual or severe reactions to any previous vaccination, history of any neurologic disorder (eg, epilepsy and autism), use of medication that influences the immune system (eg, corticosteroids), hepatitis B vaccination or any other vaccination within three months before screening (with the exception of influenza vaccinated individuals who were included), blood donation within one month before screening, administration of plasma or blood products within 12 months before screening, participation in another clinical trial within three months before screening, abnormal pre‐treatment laboratory parameters which are clinically relevant according to the investigator, bleeding disorders, participants on coumarin anticoagulants and participants receiving two or more platelet aggregation inhibitors, female subjects planning to become pregnant or breastfeeding babies until the last study visit, females with positive urine pregnancy test at screening date, excessive alcohol or illicit drug use. Excessive alcohol use was defined as >14 units per week in males and >7 units per week in females.^27^

### 2.3 Randomisation and blinding

Participants were randomly assigned to the HBAI20 vaccine or the HBVaxPro®‐10‐µg vaccine at a 3:1 ratio. Randomisation was performed by the ALEA screening and Enrolment Application Software (Formvision BV, Abcoude, the Netherlands) and with minimization of stratification factors: age (18‐25, 26‐35, 36‐50 or 51‐59 years), sex (male or female), hepatitis B vaccination history (one complete cycle or more than one cycle), and trial site (Maastricht UMC+, Antwerp University or Hospital East‐Limburg). Blinding of participants and investigators was achieved by a label that concealed the volume difference between the two vaccines. The colour was similar in both vaccines. All investigators and participants were kept blinded for the respective anti‐HBs results after the study vaccinations.

### 2.4 Procedures

The investigational vaccine (HBAI20) consisted of a new adjuvant AI20, containing 20‐µg recombinant human IL‐2 attached to 20‐µg aluminium hydroxide (CyTuVax BV, Maastricht, the Netherlands), in combination with HBVaxPro®‐10 µg.^26^ The comparator vaccine was the licensed HBVaxPro®‐10‐µg vaccine 1.0 ml (MSD VACCINS, Lyon, France). Within 1 hour before administration, the HBAI20 vaccine was prepared by an unblinded study personnel. HBVaxPro®‐10 µg was added to the AI20 adjuvant, gently mixed by inversion and aspired with a needle in one syringe (1.3 ml). Vaccines were injected into the deltoid muscle of the non‐dominant arm by an unblinded nurse following the recommended 0‐, 1‐ and 2‐month revaccination schedule for non‐responders in the Netherlands.^5^ The unblinded nurse did not perform any other study tasks.

Blood samples were drawn at screening Visit 0 (Days −30‐0), Visit 1 (Day 0), Visit 2 (Days 28‐40), Visit 3 (Days 56‐80), and Visit 4 (Days 96‐132). At the first visit (Visit 0), study participants gave written informed consent, were checked for inclusion and exclusion criteria and underwent physical examination, serological screening (complete and differential blood count, renal function, liver function, thyroid function, inflammatory parameters, HIV, hepatitis C virus [HCV] and HBV serology), and urine sampling. Urinalysis consisted of leukocytes, nitrite, pH, erythrocytes, protein and glucose measurement in all participants. A pregnancy test was taken from female participants. At Visits 2‐4, anti‐HBs antibody levels were determined, and at Visit 4, additional blood samples were drawn for haematological and biochemical evaluation. For organisational reasons, screening Visits 0 and 1 were combined in one out of three trial sites.

Participants were closely observed for 30 minutes after each vaccination for any immediate adverse events. On the day of vaccination and the subsequent 4 days, subjects were asked to record local adverse events (pain, impaired movement of injected arm, redness, swelling and induration) and systemic adverse events (fever, headache, fatigue, vomiting and diarrhoea) in individual diary cards.

### 2.5 Outcomes

The primary endpoint of this study was the immunogenicity of the HBAI20 vaccine, tested in terms of proportion of participants that have attained seroprotection at 1‐3 months after the third vaccination. Seroprotection rates measured at earlier time points and geometric mean concentrations (GMCs) were secondary endpoints.

Local and systemic adverse events were scored as absent, mild (no interference with daily activity), moderate (some interference with daily activity) or severe (prevented normal daily activities). The size of redness, swelling and induration was scored as absent, mild (>5‐25 mm), moderate (≥25‐50 mm) or severe (>50 mm). Fever was defined as oral body temperature above 37.5°C and was scored as absent, mild (37.6‐37.9°C), moderate (38.0‐38.9°C) or severe (≥39.0°C). Vomiting was scored as absent, mild (once in 24 hours), moderate (twice in 24 hours) or severe (three or more times in 24 hours). Diarrhoea was scored as absent, mild (1‐3 stools above normal), moderate (4‐5 stools above normal) or severe (>5 stools above normal). Unsolicited symptoms and serious adverse events were recorded throughout the study period. All solicited local adverse events were considered as related to the study vaccine. The investigators assessed the relationship of all other reported adverse events to vaccination.

### 2.6 Laboratory assays

Haematological, biochemical, virological parameters and urinalysis were determined using commercially available laboratory methods. Serum anti‐HBs antibody levels were analysed with the Cobas 8000 chemiluminescence assay (Roche, Germany). Subjects with anti‐HBs antibody levels ≥10 mIU/ml were considered to be seroprotected and deemed vaccine responders.^5^

### 2.7 Statistical analysis

Sample size calculation could not directly be based on phase 1 HBAI20 study data because the study population characteristics were different with respect to hepatitis B vaccination history (naive subjects vs. non‐responders). Our aim was to include between 132 and 140 subjects in agreement with other phase 2 immunogenicity and safety studies for hepatitis B vaccines.^8^, ^10^, ^14^, ^20^ Sample size for this trial was based on superiority analysis.

All data of the outcome variable anti‐HBs level were log10‐transformed. To overcome problems with 0 values, a value of +0.5 was added to all anti‐HBs levels. Categorical data were expressed as frequencies (%) and continuous variables were expressed as mean ± standard deviation (SD). For the comparison of categorical variables, either chi‐squared test or Fisher's exact test was used. Student's *t* test or Kruskall–Wallis test was used to compare groups in terms of continuous outcomes.

**FIGURE 1:**
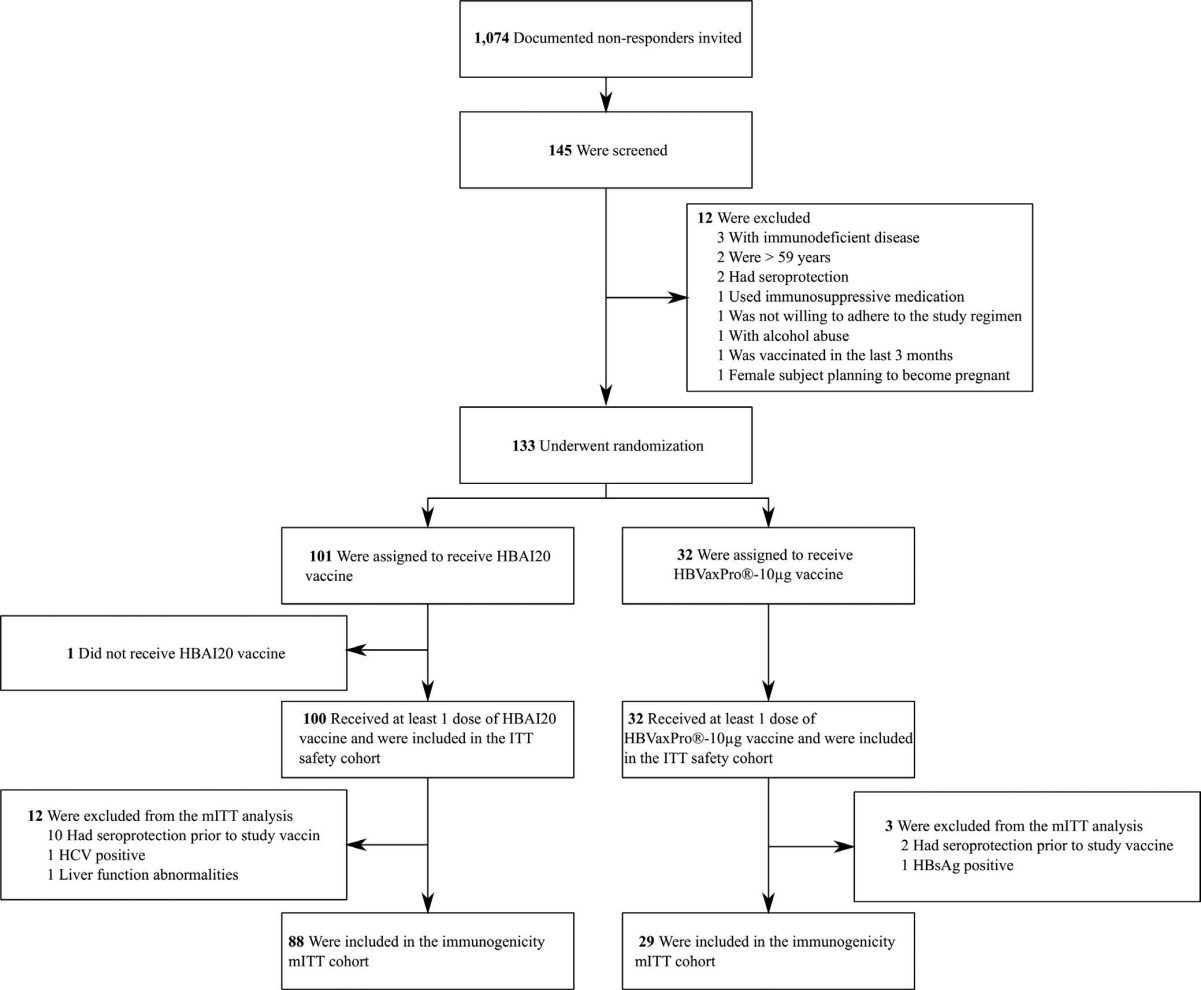
Flow chart of the study. Abbreviations: HBsAg, hepatitis B surface antigen; HCV, hepatitis C virus; ITT, intention‐to‐treat analysis; mITT, modified ITT analysis

Safety analysis was performed including all participants that received at least one vaccination (Figure 1). The percentage of adverse events for the two vaccines was compared using a chi‐squared test for the equality of proportions, or a Fisher's exact test in case of small samples. A modified intention‐to‐treat (mITT) approach was used for superiority analysis. In order to establish statistically significant superiority of the new vaccine, the one‐sided 95% confidence interval (CI) for the odds ratio (OR) of seroprotection has to be entirely above one.

Since there was one missing seroprotection outcome at Visits 3 and 4, and two at Visit 2, multiple imputation was used to impute these values. The GMC values were imputed. Multiple imputation was done using the Statistical Analysis Software (SAS Institute Inc, NC, USA) procedure PROC MI. Considering the non‐monotone pattern of missingness, the fully conditional specification (FCS) regression method was used to fill in the missing values.^28^ Imputation was done separately for the two vaccine allocations, to avoid imposing any relationship between the outcome variables and this covariate. The data were imputed five times. For each imputed dataset, a generalised linear mixed model outside the study protocol was used to estimate the vaccine effect at Visit 4, ie, 1‐3 months following the last vaccination. The stratification factors vaccination history, age, and sex were included as fixed effects in the model, in addition to vaccine allocation (ie, treatment effect). Interactions between these stratification factors and vaccine allocation were also included in the model. If not significant, the interaction effect was removed from the model, indicating no difference between vaccine allocations within the stratification group. Trial site was included as a random effect. To assess the sensitivity of the results to the method of handling missing data, a sensitivity analysis based on extreme case analyses was done, ie, worst‐case and best‐case analysis. All reported *P* values for vaccine allocation were based on one‐sided tests.

Independent data monitoring committee Clinical Trial Center Maastricht oversaw the study.

## 3 RESULTS

Participants were recruited from 5 January 2017 to 21 September 2018. The last visit occurred on 18 January 2019. Out of 1,074 persons invited, 335 HBV vaccine non‐responders replied initially for participation. A total of 41/335 (12.2%) individuals were deemed ineligible by the investigator, and 149/335 (44.5%) subjects could not be contacted after at least two attempts. In the end, 145/335 (43.3%) participants were screened, and a total of 133 subjects were enrolled and randomly assigned to receive either HBAI20 vaccine (n = 101) or HBVaxPro®‐10‐µg vaccine (n = 32).

The baseline characteristics of the 133 included subjects are shown in Table 1. The HBAI20 and HBVaxPro®‐10‐µg groups were balanced in terms of Body‐Mass Index (26 ± 4.9 vs. 26 ± 6.5, *P* = .543) and the number of individuals with undetectable anti‐HBs levels (<2 mIU/ml) prior to study vaccination (69/101 [68.3%] vs. 27/32 [84.4%], *P* = .124).

**TABLE 1:** Baseline characteristics of the subjects

Characteristic	HBAI20 group (n = 101)	HBVaxPro®‐10‐µg group (n = 32)
Mean age, years	40 ± 14.3	40 ± 14.8
Sex, no. (%)		
Male	42 (41.6%)	13 (40.6%)
Female	59 (58.4%)	19 (59.4%)
Hepatitis B vaccination, no. (%)		
1 complete cycle	46 (45.5%)	14 (43.8%)
More than 1 cycle	55 (54.5%)	18 (56.2%)

Of the 133 randomised participants, 132 received at least one dose of HBAI20 vaccine or HBVaxPro®‐10‐µg vaccine. There were 117 participants included in the mITT immunogenicity analysis; 15 subjects were excluded, mainly due to seroprotection attained prior to study vaccination (Figure 1). Interval between last hepatitis B vaccination and study entry was not associated with seroprotection prior to study vaccination: seroprotection was 5.4% (3/56 participants with an interval of ≤1 year between previous vaccination and study entry) versus 11.7% (9/77 participants with an interval of >1 year between previous hepatitis B vaccination and enrolment into the study), *P* = .208.

File S1 shows the baseline characteristics of the 117 subjects included in the mITT analysis. The baseline characteristics of the HBAI20 group included in the mITT analysis were similar to those in the HBVaxPro®‐10‐µg group. A total of 116 participants received all three doses (one drop‐out in the HBAI20 group due to an adverse event after one vaccination).

**FIGURE 2:**
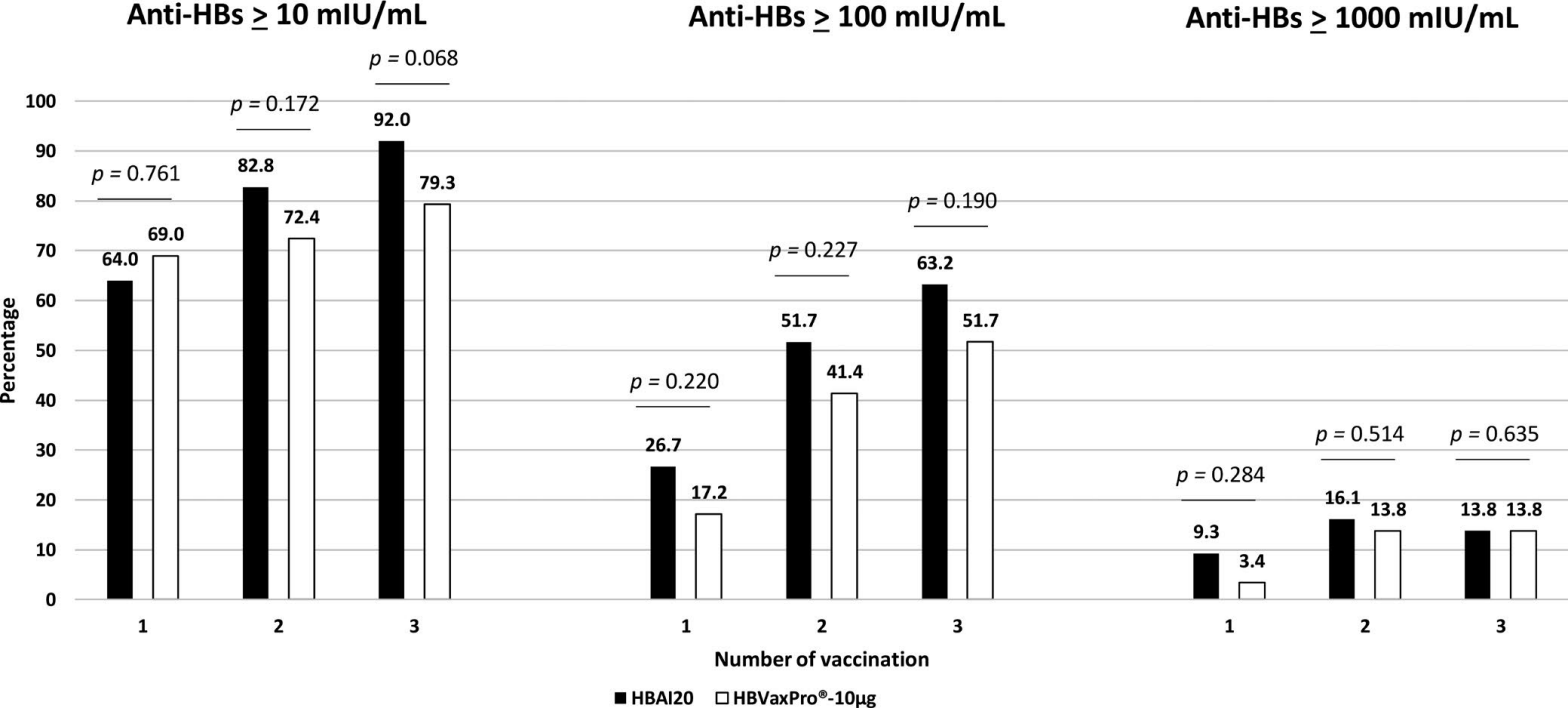
Percentage of individuals in HBAI20 and HBVaxPro®‐10‐µg group with hepatitis B surface antibody levels ≥10, ≥100 and ≥1,000 mIU/ml after one, two and three vaccinations. n = 86 for first HBAI20 vaccine and n = 87 for second and third HBAI20 vaccine. n = 29 for first, second and third HBVaxPro®‐10µ‐g vaccine

A total of 80 (92.0%) participants in the HBAI20 group and 23 (79.3%) participants in the HBVaxPro®‐10‐µg group attained seroprotection, as measured at 1‐3 months after the third vaccination, *P* = .068 (Figure 2). Seven participants did not respond to the new HBAI20 vaccine and their characteristics are illustrated in File S2. Comparison of baseline characteristics between non‐responders and responders to HBAI20 vaccine indicated that non‐responders were older (50 ± 11.2 years vs. 40 ± 14.3 years, *P* = .040; File S3). Six participants did not respond to the HBVaxPro®‐10‐µg vaccine and a comparison of their baseline characteristics to the 23 responders indicated that non‐responders were older (49 ± 9.1 year vs. 37 ± 15.0, *P* = .067, File S4).

One month after first vaccination, seroprotection percentages were respectively 64.0% vs. 69.0% (*P* = .761), and these numbers were 82.8% vs. 72.4% (*P* = .172) at 1 month after second vaccination (Figure 2).

One to 3 months following the last vaccination, 55 participants (63.2%) in the HBAI20 group and 15 (51.7%) in the HBVaxPro®‐10‐µg group had anti‐HBs levels ≥100 mIU/ml (*P* = .190). Figure 2 illustrates the proportion of individuals with anti‐HBs levels ≥10, ≥100 and ≥1,000 mIU/ml after one, two and three vaccinations with HBAI20 or HBVaxPro®‐10‐µg vaccine.

The GMCs were higher in the HBAI20 group than in the HBVaxPro®‐10‐µg group after vaccination with one (21.3 ± 1.7 vs. 18.3 ± 2.1, *P* = .390), two (72.3 ± 1.4 vs. 41.5 ± 2.2, *P* = .157) and three study vaccines (161.4 ± 0.9 vs. 65.0 ± 2.0, *P* = .037).

Sixty‐four of 117 subjects had received more than one hepatitis B vaccination cycle prior to inclusion in the present study and the seroprotection percentages were as follows: 66.0% vs. 64.7% (*P* = .575), 80.9% vs. 70.6% (*P* = .289) and 87.2% vs. 70.6% (*P* = .120) after respectively one, two and three vaccinations with HBAI20 and HBVaxPro®‐10‐µg vaccine.

Using a generalised linear mixed model, adjusting for stratification factors, the primary endpoint was met, and a higher odds of seroprotection with the HBAI20 vaccine was shown (adjusted OR = 3.48, lower bound of 95% CI = 1.19, *P* = .028) (Table 2). Since imputation of one missing value at visit 4, ie, 1‐3 months after the third vaccination, resulted in a seroprotected score, results from Table 2 correspond to the best‐case scenario. For the worst‐case scenario, ie, person with missing value did not achieve seroprotection, HBAI20 vaccine was still shown to give a higher odds of seroprotection (adjusted OR = 2.95, lower bound of 95% CI = 1.05, *P* = .042).

**TABLE 2:** Results of the generalised linear mixed model

Characteristic	Estimate (SE)	OR (95% CI)	*P* value
HBAI20 vaccine	1.25 (0.65)	3.48 (1.19 to ∞)	.028
Age (≥40 years)	−0.88 (0.73)	0.42 (0.10‐1.76)	.231
Male sex	−0.47 (0.64)	0.62 (0.18‐2.19)	.459
More than 1 hepatitis B vaccination cycle	−1.60 (0.82)	0.20 (0.04‐1.03)	.054

Note

Age, sex and hepatitis B vaccination history were included as fixed effects in the model, in addition to vaccine allocation, ie, treatment effect. Trial site was included as a random effect.

Abbreviations: CI, confidence interval; OR, odds ratio; SE, standard error.

Within the group of individuals who received the HBAI20 vaccine, seroprotection after three vaccinations was seen in 11 (73.3%) out of 15 participants without pain at the injection site, compared to 76 (96.2%) out of 79 participants with pain at the injection site, *P* = .012. In the HBVaxPro®‐10‐µg group, no association was found between pain at the injection site and seroprotection after three vaccinations (6/10 [60.0%] without pain at the injection site vs. 18/20 (90.0%) with pain at the injection site, *P* = .141).

**FIGURE 3:**
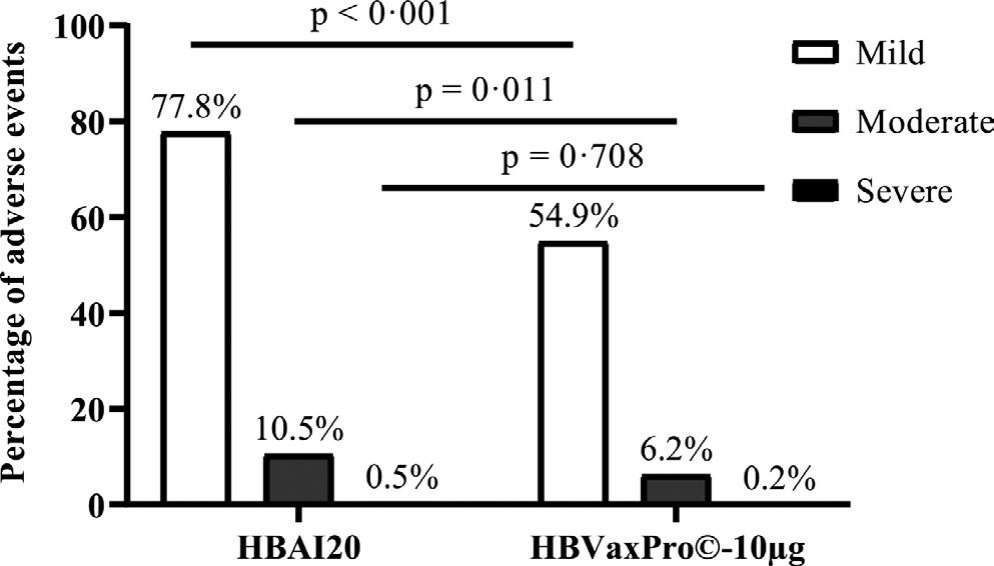
Percentage of mild, moderate and severe adverse events (local and systemic) at the day of vaccination and the 4 consecutive days after three vaccinations with the HBAI20 vaccine or the HBVaxPro®‐10‐µg vaccine

There was a higher incidence of solicited local adverse events in the HBAI20 group than HBVaxPro®‐10‐µg group (13.7% vs. 8.7%, *P* < .001), while the overall incidence of systemic adverse events was similar for both vaccines (4.0% vs. 3.4%, *P* = .198). The percentage of participants with mild, moderate and severe adverse events (local and systemic) in each group is illustrated in Figure 3.

The most frequently reported local adverse event in both the HBAI20 group (32.7%) and HBVaxPro®‐10‐µg group (18.6%) was pain at the injection site, *P* < .001. Pain at the injection site resolved within 4 days after vaccination for 94% and 97% of participants, respectively. There were no severe local adverse events in the HBVaxPro®‐10‐µg group, and these were low in the HBAI20 group. None of the participants experienced severe local adverse events for longer than 4 days. The frequencies of any and severe local adverse events are shown in Table 3.

**TABLE 3:** Incidence of local solicited adverse events during the day of vaccination and each of the consecutive 4 days after the first, second and third vaccination

Local adverse events		HBAI20 (n = 1,345 days)	HBVaxPro®‐10 µg (n = 435 days)	*P* value
Pain at the injection site	Any	440 (32.7%)	81 (18.6%)	<.001
	Severe	0 (0.0%)	0 (0.0%)	—
Impaired movement of injected arm	Any	198 (14.7%)	43 (9.9%)	.013
	Severe	0 (0.0%)	0 (0.0%)	—
Redness	Any	95 (7.06%)	16 (3.7%)	.015
	Severe	2 (0.1%)	0 (0.0%)	1.000
Swelling	Any	79 (5.9%)	25 (5.8%)	1.000
	Severe	1 (0.1%)	0 (0.0%)	1.000
Induration	Any	106 (7.9%)	24 (5.5%)	.123
	Severe	1 (0.1%)	0 (0.0%)	1.000

The most frequently reported systemic adverse event was fatigue (10.6% vs. 9.7%, *P* = .655), which resolved within 4‐day post‐vaccination in 96% and 91% of the participants in the HBAI20 group and HBVaxPro®‐10‐µg group, respectively. The incidence of severe systemic adverse events was low and comparable in both groups. In all cases, severe systemic adverse events resolved within 4 days. Table 4 lists the overall incidence of systemic adverse events.

**TABLE 4:** Incidence of systemic solicited adverse events during the day of vaccination and each of the consecutive 4 days after the first, second and third vaccination

Systemic adverse events		HBAI20 (n = 1,345 days)	HBVaxPro©‐10 µg (n = 435 days)	*P* value
Fever	Any	10 (0.7%)	2 (0.5%)	.771
	Severe	1 (0.1%)	1 (0.2%)	.985
Headache	Any	89 (6.6%)	25 (5.7%)	.595
	Severe	0 (0.0%)	1 (0.2%)	.552
Fatigue	Any	142 (10.6%)	42 (9.7%)	.655
	Severe	2 (0.1%)	0 (0.0%)	1.000
Vomiting	Any	1 (0.1%)	1 (0.2%)	.985
	Severe	0 (0.0%)	0 (0.0%)	—
Diarrhoea	Any	28 (2.1%)	4 (0.9%)	.168
	Severe	0 (0.0%)	0 (0.0%)	—

Unsolicited symptoms were reported by 58 (57.4%) subjects receiving HBAI20 vaccine and by 18 (56.3%) subjects receiving HBVaxPro®‐10 µg, *P* = .210. Six (5.9%) individuals in the HBAI20 group and four (12.5%) individuals in the HBVaxPro®‐10 µg had increased alanine aminotransferase values (>ULN) after intervention, *P* = .220. There were 10 (9.9%) cases of asymptomatic urinary tract infection in healthy non‐pregnant women receiving HBAI20 vaccine; this number was zero (0.0%) in those receiving HBVaxPro®‐10‐µg vaccine, *P* = .250. No medically significant abnormalities were observed in haematological, renal and thyroid parameters. Two serious adverse events were reported during the study period, one in the HBAI20 group (ie, hernia nuclei pulposi) and one in the HBVaxPro®‐10 µg (ie, epileptic seizure). Both were considered to be unrelated to vaccination by the investigators.

## 4 DISCUSSION

Hepatitis B vaccine non‐responders, ie, individuals with a deficient antibody response after three or more vaccinations, represent an unresolved problem in hepatitis B vaccination.^11^ In order to reach hepatitis B elimination by 2030 as advocated by the World Health Organization, it is important to establish protective anti‐HBs levels also in hepatitis B vaccine non‐responders, who represent 5%–10% of the vaccinated adult population.^11^, ^29^, ^30^

After adjusting the analysis for stratification factors, this phase 2 trial demonstrated that immunogenicity of the investigational HBAI20 vaccine was superior to that of the licensed HBVaxPro®‐10‐µg vaccine, when given to healthy hepatitis B vaccine non‐responders at 0, 1 and 2‐month schedule. Safety was comparable between both vaccines.

Seroprotection rates in our comparator arm HBVaxPro®‐10 µg were higher compared to previous studies.^6^, ^7^, ^8^, ^9^, ^10^ Previous studies enrolling hepatitis B vaccine non‐responders have found that three revaccination doses with a licensed hepatitis B vaccine induced seroprotection in 50%–69% of the individuals.^6^, ^7^, ^8^, ^9^, ^10^ In our study, three revaccinations with HBVaxPro®‐10 µg induced protective anti‐HBs levels in 79% of the non‐responders. These variations can be explained by the use of different licensed vaccines (eg, Engerix‐B®, Recombivax HB®) and distinct study populations with different definitions of non‐responder.^8^, ^10^, ^17^, ^31^

The primary endpoint of this study was met: compared to HBVaxPro®‐10 µg, subjects receiving HBAI20 vaccine were about 3.5 times more likely to be seroprotected around 1‐3 months following the last vaccination. The observed seroprotection percentage after three vaccinations with HBAI20 vaccine was 92%, which is higher than expected in this population of hepatitis B vaccine non‐responders. Moreover, seroprotection after two doses of HBAI20 vaccine was comparable to those attained after three doses of the licensed HBVaxPro®‐10‐µg vaccine in this healthy non‐responder population. It would be worth to investigate whether HBAI20 vaccine could provide an accelerated antibody response compared to the control vaccine in travellers to intermediate or high HBV endemic areas and those with high‐risk behaviour (eg, sex workers) as this could represent an important benefit.

One should acknowledge that the difference in seroprotection between both vaccines in this study is only linked with the adjuvant AI20.^26^ AI20 is a cytokine‐based adjuvant consisting of 20‐µg recombinant human IL‐2 attached to 20‐µg aluminium hydroxide.^26^ IL‐2 is critical in promoting generation of T helper 1 (Th1) and Th2 cells, while IL‐2 also improves dendritic cell‐mediated stimulation of allogeneic CD4 T cell proliferation.^26^, ^32^, ^33^, ^34^, ^35^ Prior studies assessing the properties of IL‐2‐inducing hepatitis B‐specific immune response have been limited by the short half‐life of IL‐2.^34^, ^36^, ^37^, ^38^ The IL‐2 in the new AI20 adjuvant is adsorbed to aluminium hydroxide, facilitating the slow release of highly concentrated IL‐2 nano‐aggregates. Taking into account the additional immunogenic properties of the AI20 adjuvant, it might be reasonable to consider the AI20 adjuvant for other vaccines conferring a suboptimal response, such as for tuberculosis vaccines.

Seven hepatitis B vaccine non‐responders did not respond to the novel HBAI20 hepatitis B vaccine. Although single‐nucleotide polymorphisms in IL‐2 region are associated to a number of autoimmune diseases, further research is warranted to assess the association between IL‐2 gene polymorphisms and the risk of non‐response to hepatitis B vaccination.^39^, ^40^

Safety data from this phase 2 study indicate comparable rates of systemic solicited adverse events, unsolicited symptoms and serious adverse events, except for significantly higher rates of local adverse events within the HBAI20 group. The majority of the adverse events resolved within 5 days and only a few adverse events were reported as severe. The higher incidence of local adverse events could mainly be attributed to pain at the injection site which was reported by 33% in HBAI20 group and 19% in HBVaxPro®‐10‐µg group, respectively, and was similar to those reported in previous studies with adjuvanted hepatitis B vaccines.^8^, ^10^ With respect to local adverse events, we could also show greater immunity, ie, seroprotection, in those participants with pain at the injection site in the HBAI20 group. Further research is warranted on whether the presence of pain at the injection site is associated with more attraction of innate cells to the inoculum and subsequent higher seroprotection rate.

This study had some limitations. First, a formal sample size calculation could not be performed since the naive study population in phase 1 study differed significantly from the non‐responder population in the current study. Therefore, this study was underpowered to find significant associations between HBAI20 vaccine and certain study endpoints in univariate analyses. However, univariate analyses do not account for heterogeneity between trial sites, and the baseline characteristics are almost never completely similar between both study groups, even after randomisation. By adjusting for stratification factors, the results from the multiple models avoid an unnecessary loss in power and are more reliable than those obtained from univariate analyses. In that respect, superiority of HBAI20 vaccine was shown in the multiple model. Second, our study was limited by its inability to account for other risk factors, such as smoking behaviour and HLA DQ2 phenotype. Third, although non‐responder in the current study was rigorously defined in line with the Advisory Committee on Immunization Practices, 12 out of 133 randomised non‐responders appeared to be already seroprotected before study vaccination.^5^ This might be related to incomplete documentation of hepatitis B vaccination schedule. The current study did not adopt a restriction related to the interval between prior hepatitis B vaccination and inclusion into the study. Our definition of non‐responder was in that respect similar to the two single‐blinded, randomised controlled trials in healthy non‐responders, one with Fendrix® and one with Heplisav‐B®.^8^, ^10^ Fourth, a 1:1 randomisation as desired in many vaccine studies was not approved by the local ethics committee considering the expected low seroprotection rate with standard hepatitis B vaccines among our study population of non‐responders. Subsequently, the 3:1 randomisation in the current study might have resulted in small proportions of better responders in the control group skewing the results.

In conclusion, these results are suggestive of increased immunogenicity of the HBAI20 vaccine among healthy non‐responders to three or more hepatitis B vaccinations, in addition to confirming that the HBAI20 vaccine could be safely administered. These results support a phase 3 study to assess the benefits of the HBAI20 vaccine in the unresolved group of healthy non‐responders. It would be worth to investigate the benefits of the HBAI20 vaccine in other risk groups for a suboptimal immune response, ie, patients with advanced liver disease, haemodialysis patients, and patients with other immunodeficient conditions.

## CONFLICT OF INTEREST

ÖMK received travel grants from Gilead Sciences and his institution received grants from Gilead Sciences, AbbVie, MSD and CyTuVax BV GR has received research grants from AbbVie, MSD, Janssen Pharmaceuticals, and has acted as a consultant/advisor for AbbVie, MSD, Gilead Sciences and Bristol‐Myers Squibb. PVD acts as chief and principal investigator for vaccine trials conducted on behalf of the University of Antwerp, for which the University obtains research grants from vaccine manufacturers; speakers fees for presentations on vaccines are paid directly to an educational fund held by the University of Antwerp. PVD receives no personal remuneration for this work. JA is an employee of CyTuVax BV FF is inventor of the use of cytokine macro‐aggregates as adjuvant and is co‐founder and shareholder of CyTuVax BV AOL received honorarium for lectures from GSK and Janssen‐Cilag, all payments were invoiced by the Department of Medical Microbiology, Maastricht UMC+. NH is holder of the chair in evidence‐based vaccinology supported though a gift by Pfizer. All outside the submitted work. The following authors reported that they have no conflicts of interest: CK, PDS and PS.

## AUTHOR CONTRIBUTIONS

ÖMK, PDS, GR, PVD, JA, FF, PS and AOL contributed to the conception and design of the study. GR, PVD, PS and AOL supervised ÖMK and PDS to collect data. Following statistical analysis of data by CK and NH, ÖMK drafted the first version of the paper; the co‐authors critically revised the article and approved the final version to be submitted, including the authorship list.

## ETHICS APPROVAL

The protocol was approved by the local ethics committee and was conducted in accordance with the guidelines of the Declaration of Helsinki and its amendments and in accordance with good clinical practice and local laws.

## CLINICAL TRIAL NUMBER

ClinicalTrials.gov number, NCT03415672.

## Supporting information

File S1‐S4

## Data Availability

Proposals should be directed to the corresponding author; to gain access, data requestors will need to sign a data access agreement. Only individual participant data that underlie the results reported in this article, after de‐identification, will be shared.
