# Supplementary material for: Immunogenicity and safety of HBAI20 Hepatitis B vaccine in non‐responders: Double‐blinded, randomised, controlled phase 2 trial
Source: Liver Int. 2021 May 24;41(10):2318–27. doi: 10.1111/liv.14939 (PMC8518051; doi:10.1111/liv.14939)
Supplement: Supplementary file 1 — File S1‐S4 [file LIV-41-2318-s001.docx]

**Immunogenicity and safety of HBAI20 Hepatitis B vaccine in healthy non-responders: double-blinded, randomised, controlled phase 2 trial.**

Özgür Muhammet Koc, Philippe De Smedt, Cécile Kremer, Geert Robaeys, Pierre Van Damme, Niel Hens, Jorge Almeida, Frank Falkenberg, Paul Savelkoul, Astrid Oude Lashof, and the BE RESPONDER Study Group

| **Supplementary file S1 Baseline characteristics of the subjects included in the modified intention to treat analysis (n = 117).** | | | |
| --- | --- | --- | --- |
| **Characteristic** | **HBAI20**  **Group**  **(n=88)** | **HBVaxPro**®**-10µg**  **Group**  **(n=29)** | **P value** |
| Mean age – years | 40 + 14.3 | 40 + 14.7 | 0.810 |
| Sex– no. (%)  Male  Female | 37 (42.1%)  51 (57.9%) | 11 (37.9%)  18 (62.1%) | 0.863 |
| Mean BMI – kg/m^2^ | 26 ± 5.1 | 26 ± 6.7 | 0.439 |
| Geometric mean concentrations – mIU/mL | 1.0 ± 2.0 | 0.5 ± 1.4 | 0.226 |
| Hepatitis B vaccination – no. (%)  1 complete cycle  More than 1 cycle | 41 (46.6%)  47 (53.4%) | 12 (41.4%)  17 (58.6%) | 0.784 |

^Abbreviations: BMI: Body-Mass Index; Anti-HBs: hepatitis B surface antibodies.^

# **Supplementary file S2 Characteristics of seven non-responders to new HBAI20 hepatitis B vaccine**

| **Patient** | **Sex** | **Age > 40 years** | **BMI** | **Baseline anti-HBs level (mIU/mL)** | **Number of hepatitis B vaccinations prior to study** |
| --- | --- | --- | --- | --- | --- |
| *Pt 1* | Female | No | 24 | 0 | 7 |
| *Pt 2* | Male | Yes | 30 | 0 | 6 |
| *Pt 3* | Male | Yes | 34 | 0 | 6 |
| *Pt 4* | Female | Yes | 34 | 0 | 6 |
| *Pt 5* | Male | Yes | 26 | 2.3 | 6 |
| *Pt 6* | Male | Yes | 26 | 0 | 6 |
| *Pt 7* | Female | Yes | 26 | 0 | 4 |

^Abbreviations: BMI: Body-Mass Index; anti-HBs: hepatitis B surface antibodies; Pt: patients.^

| **Supplementary file S3 Comparison of baseline characteristics between non-responders and responders to HBAI20 vaccine.** | | | |
| --- | --- | --- | --- |
| **Characteristic** | **Non-responders**  **(n=7)** | **Responders**  **(n=80)** | **P value** |
| Mean age – years | 50 ± 11.2 | 40 ± 14.3 | 0.040 |
| Sex– no. (%)  Male  Female | 4 (57.1%)  3 (42.9%) | 33 (41.3%)  47 (58.7%) | 0.452 |
| Mean BMI – kg/m^2^ | 29 ± 4.3 | 26 ± 4.9 | 0.147 |
| Geometric mean concentrations – mIU/mL | 0.3 ± 0.9 | 1.0 ± 2.1 | 0.467 |
| Hepatitis B vaccination – no. (%)  1 complete cycle  More than 1 cycle | 1 (14.3%)  6 (85.7%) | 39 (48.7%)  41 (51.3%) | 0.118 |

^Abbreviations: BMI: Body-Mass Index; anti-HBs: hepatitis B surface antibodies.^

| **Supplementary file S4 Comparison of baseline characteristics between non-responders and responders to HBVaxPro®-10µg vaccine.** | | | |
| --- | --- | --- | --- |
| **Characteristic** | **Non-responders**  **(n=6)** | **Responders**  **(n=23)** | **P value** |
| Mean age – years | 49 ± 9.1 | 37 ± 15.0 | 0.067 |
| Sex– no. (%)  Male  Female | 2 (33.3%)  4 (66.7%) | 9 (39.1%)  14 (60.9%) | 1.000 |
| Mean BMI – kg/m^2^ | 23.7 ± 1.5 | 26.8 ± 7.4 | 0.306 |
| Geometric mean concentrations – mIU/mL | 0.9 ± 1.5 | 0.4 ± 1.4 | 0.138 |
| Hepatitis B vaccination – no. (%)  1 complete cycle  More than 1 cycle | 1 (16.7%)  5 (83.3%) | 11 (47.8%)  12 (52.2%) | 0.354 |

^Abbreviations: BMI: Body-Mass Index; anti-HBs: hepatitis B surface antibodies.^
